# Substantial increase in perceived benefits over harms of COVID-19 outbreak but persistent socioeconomic disparities: Comparison of two cross-sectional surveys in Hong Kong from 2020 to 2021

**DOI:** 10.3389/fpubh.2022.1012146

**Published:** 2022-11-17

**Authors:** Hiu Tin Leung, Wei Jie Gong, Shirley Man Man Sit, Agnes Yuen Kwan Lai, Sai Yin Ho, Man Ping Wang, Tai Hing Lam

**Affiliations:** ^1^School of Public Health, The University of Hong Kong, Hong Kong, Hong Kong SAR, China; ^2^Department of General Practice, Health Science Center, Shenzhen University, Shenzhen, China; ^3^School of Nursing, The University of Hong Kong, Hong Kong, Hong Kong SAR, China

**Keywords:** COVID-19, perceived benefits, perceived harms, socioeconomic disparities, cross-sectional study

## Abstract

**Background:**

We have reported both perceived benefits and harms of the COVID-19 outbreak and their socioeconomic disparities amid the pandemic in Hong Kong. We further investigated whether such perceptions and disparities had changed after 10 months.

**Methods:**

Under the Hong Kong Jockey Club SMART Family-Link Project, we conducted two cross-sectional surveys online on perceived personal and family benefits and harms of the COVID-19 outbreak in Hong Kong adults in May 2020 (after Wave 2 was under control; *N* = 4,891) and in February and March 2021 (after Wave 4 was under control; *N* = 6,013). We collected sociodemographic information, including sex, age, education, household income, and housing. Using multivariate models of analysis of covariance (MANCOVA), we compared perceived benefits and harms and socioeconomic disparities between the two surveys.

**Results:**

Adjusting for sex and age, the prevalence of 17 out of 18 perceived personal and family benefits of COVID-19 outbreak increased (Ps < 0.001). Six of 11 perceived personal and family harms decreased (Ps < 0.001) and 4 increased (Ps < 0.001). The total number of perceived personal and family benefits increased substantially (Ps < 0.001), whereas that of perceived personal harms decreased (*P* = 0.01) and family harms remained stable (*P* > 0.05). Socioeconomic disparities, however, persisted—more perceived benefits in those with higher socioeconomic status (Ps < 0.001) and more perceived harms in those with lower (Ps ≤ 0.005).

**Conclusion:**

We have first reported that perceived personal and family benefits of the COVID-19 outbreak increased substantially over 10 months amid the pandemic, while perceived personal and family harms were lower and stable, respectively. Socioeconomic disparities of the perceived benefits and harms persisted, which need to be monitored and addressed urgently.

## Introduction

The coronavirus disease 2019 (COVID-19) poses one of the greatest global public health crises in recent history. Apart from its dire effects on physical health, depression and anxiety have also surged amid the pandemic globally (1). The Organization for Economic Co-operation and Development (OECD) unemployment rate increased by 3% points, reaching 8.8% at the onset of the crisis (2). However, the pandemic may have some unintended benefits. With the stringent public health measures, cold and flu cases plummeted worldwide (3, 4). Information and communications technology has made work-from-home possible, particularly for professionals, in developed countries (5). In addition to reducing risks of infection, such work-from-home arrangements could also benefit workers' health and well-being as well as their family and interpersonal relationships (6).

In Hong Kong, we have reported the mental health crisis early amid the COVID-19 pandemic (7) and factors associated with mental health symptoms (8). The levels of probable depression and anxiety declined further into the pandemic (9), and seasonal flu had also subsided abruptly (10). Despite the economic slowdown in general, logistics related sectors, such as those providing postal and courier services, have seen business increased by over 30% (11).

The impact of COVID-19 showed remarkable socioeconomic disparities. Across the globe, lower socioeconomic status is linked to less frequent COVID-19 testing, more positive tests, and more hospitalization and deaths (12–16). Amid the COVID-19 pandemic, people of lower socioeconomic status or those who reported greater economic hardship showed higher depression, anxiety and stress and lower psychological well-being (17–20). Relatedly, low-paying jobs also saw a much larger decrease in paid work hours during the pandemic than high-paying jobs (2). In addition, workers with higher qualifications and those who worked in larger firms were more able to work from home (5). Consistent with these findings, we found that Hong Kong people with higher socioeconomic status reported more perceived benefits, whereas those with lower socioeconomic status reported more perceived harms from the COVID-19 outbreak (21). Our search of PubMed using the keywords “benefits” and “harms” and “socioeconomic” and “COVID-19” up to 5 June 2022 yielded seven reports (excluding our own) that investigated both potential benefits and harms of COVID-19 (6, 22–27). However, these reports were based largely on qualitative data or casual observations; none directly compared both perceived benefits and harms and their socioeconomic disparities between early and later waves of outbreak amid the pandemic.

Perception of the COVID-19 pandemic could change over time. As mass vaccination programme is made available, more and more people are protected against serious consequences of COVID-19 (28, 29). People could also become emotionally adapted to the pandemic (9, 30) through cognitive reappraisal where adverse events are seen as positive challenges rather than merely threats (31). However, socioeconomic disparities could persist despite changes in the perception of the pandemic. Flexible work arrangements remain largely irrelevant to manual workers; grassroot families are still less capable to cope with lowering income; cross-infections are still more likely in crowded homes. People's happiness and well-being are linked to their socioeconomic status relative to others as well as their absolute status (32). Such social comparison may affect various domains in life, breeding various forms of socioeconomic disparities (16, 33, 34), especially in Hong Kong, where income inequality is among the highest in the world (35).

**Figure 1:**
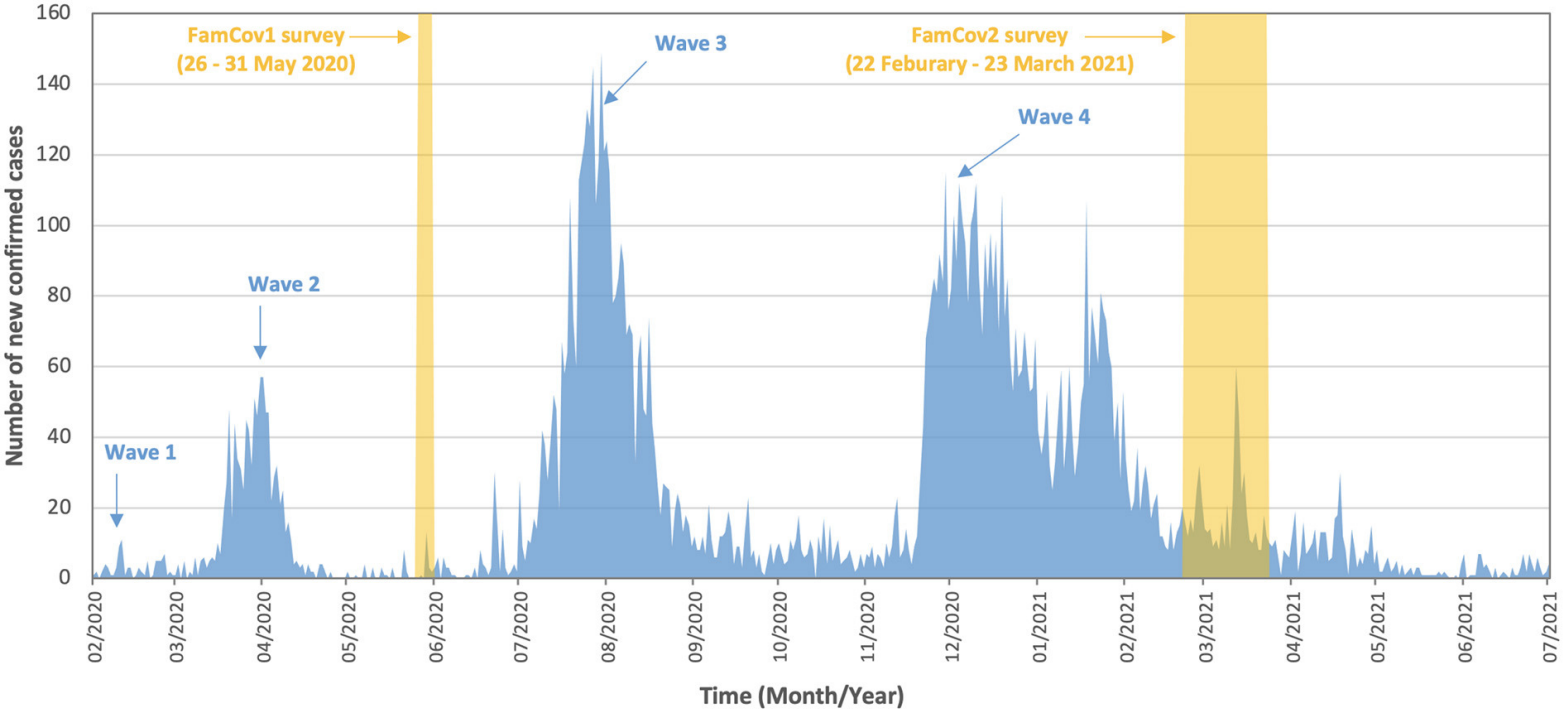
Number of new confirmed cases of COVID-19 from February 2020 to July 2021 in Hong Kong. The highlighted periods show the time period during which data of FamCov1 and FamCov2 surveys were collected. Wave 5 started in Jan 2022 with a peak of 56,827 cases (almost all Omicron BA.2) on 3 March 2022, and reduced to 234 cases on 16 May 2022.

We previously published on both perceived benefits and harms of the COVID-19 outbreak and their socioeconomic disparities amid the pandemic. The data were derived from our FamCov1 population survey on Hong Kong adults when Wave 2 was under control in May 2020 (21, 36, 37). At that time, the prevalence of perceived benefits was lower than that of perceived harms, but no other reports were available for comparison. The pandemic then continued with the more serious Wave 3 (July–August 2020) and subsequently a more prolonged Wave 4 (November 2020–March 2021; Figure 1). From February to March 2021, after Wave 4 was under control, we conducted a second population survey (FamCov2) to re-assess the perceived benefits and harms and to measure changes. In the present paper, we compared the perceived benefits and harms of the COVID-19 outbreak and their relations with socioeconomic status in the two surveys (i.e., across these two time points) on Hong Kong adults.

## Subjects and methods

### Samples and procedures

The data were collected from two surveys, FamCov1 and FamCov2, under the Hong Kong Jockey Club SMART Family-Link Project. Details of FamCov1 were described previously (21, 36, 37). Briefly, FamCov1 was a population-based cross-sectional online survey conducted from 26–31 May 2020. The participants were Hong Kong Chinese residents aged 18 years or above. The target respondents were given information on the purpose of the survey, with emphasis on confidentiality. The questionnaire (in Chinese) was developed by our project team. The Hong Kong Public Opinion Research Institute (HKPORI), a well-known local survey agency, was commissioned to conduct the survey. The online questionnaire was anonymous and self-administered. HKPORI reviewed the questionnaire, tested it in a pilot survey and found no problems. All data were collected on the e-platform. A total of 70,984 invitation emails were sent by HKPORI to its probability—and non-probability-based—panels; 20,103 emails were opened and 4,944 participants responded. Because our target population was Hong Kong adults having at least one family member, we excluded those having no family members (*n* = 30) and having more than 30% missing values (*n* = 23), leaving 4,891 participants who provided useable data.

FamCov2 was the second population-based cross-sectional survey conducted from 22 February to 23 March 2021. The questionnaire (in Chinese) was developed by our project team, based on that of FamCov1. The methods were similar to those of FamCov1. Briefly, email invitation was sent to 95,705 adults with valid email addresses from the panel. 48,825 (51.0%) invitation emails were opened, and 6,013 respondents (12.3% of 48,825) successfully completed the survey. Both surveys were designed to include as many respondents as possible within a short period at low cost.

Ethics approval was obtained from the Institutional Review Board of the University of Hong Kong/Hospital Authority Hong Kong West Cluster (IRB reference no.: UW20-238).

### Measures

Perceived personal and family benefits and harms of COVID-19 outbreak were assessed, with the questions, “What benefits or harms have the COVID-19 outbreak brought to you/your family?”, each followed by a list of putative perceived benefits and harms of COVID-19 based on literature review and team discussion. The present analysis was on data of common items (answer options: yes/no) used in both FamCov1 and FamCov2, which were 10 perceived personal benefits, 8 perceived family benefits, 5 perceived personal harms, and 6 perceived family harms (Table 2). These items were categorized into three domains: (a) physical, (b) psychological, and (c) social. Based on participants' responses of yes or no (1/0) on each item, we summed the “yes” responses to obtain the total scores for perceived personal benefits (10 items, score range 0–10), perceived family benefits (8 items, score range 0–8), perceived personal harms (5 items, score range 0–5), and perceived family harms (6 items, score range 0–6).

Sociodemographic information was obtained, including sex, age, educational attainment, household monthly income, and housing type. Sociodemographic variables were coded as follows: age into six groups (18–24, 25–34, 35–44, 45–54, and 55–64, 65 years or above), education into two groups (secondary or lower, tertiary), household income into three groups (HKD 19,999 or below, HKD 20,000–39,999, HKD 40,000 or above; US$1 = HK$7.8), and housing into two groups (rented, owned). A socioeconomic status (SES) score ranged 0–3 was calculated by summing scores from education (0 = secondary or lower, 1 = tertiary), household monthly income per person (0 = below or equal to median, 1 = above median), and housing (0 = rented, 1 = owned). An SES score of zero was labeled as “very low”, 1 as “low”, 2 as “medium”, and 3 as “high”. We have previously used this classification of socioeconomic status and showed that it was positively associated with perceived benefits and negatively associated with perceived harms (21).

### Statistical analysis

All statistical analyses were conducted with IBM SPSS v. 28 and *P* < 0.05 was considered statistically significant. To improve the representativeness of the sample, all data were weighted using random iterative method (RIM) weighting based on sex, age, and education distribution of the Hong Kong general adult population in 2020 (38). Descriptive statistics were used for the sociodemographic characteristics of the participants. Chi-squared tests were used to examine sociodemographic differences across the two surveys and Cohen's W (0.10, small; 0.30, medium; 0.50, large) were used to estimate the effect sizes of these differences.

A multivariate model of analysis of covariance (MANCOVA) was used to estimate the weighted prevalence and 95% confidence intervals of each item of perceived benefits and harms. The effects of sex and age were adjusted for by entering these variables as covariates. All individual perceived benefits and harms items were entered as the dependent variables and the time of the surveys (FamCov2 and FamCov1) was entered as an independent variable. Prevalence ratios (PRs) were calculated by dividing the prevalence of each item obtained in FamCov2 by the prevalence of the same item in FamCov1. Statistical effect sizes were estimated using partial eta squared (0.01, small; 0.06, medium; 0.14, large).

MANCOVA was used to test and provide the estimated marginal means (with 95% confidence intervals) of the total number of perceived personal benefits, perceived family benefits, perceived personal harms, and perceived family harms. Each of these four total scores were entered as dependent variables in the model. The time of the surveys (FamCov2 and FamCov1) as well as the SES scores (0–3) were entered as independent variables. The effects of sex and age were adjusted for by entering these variables as covariates.

## Results

### Participants' characteristics in FamCov1 and FamCov2

From May 26 to 31, 2020, FamCov1 survey collected usable data from 4,891 adults. From February 22 to March 23, 2021, FamCov2 survey collected usable data from 6,013 adults. Table 1 shows, after weighting, similar percentages of women (52.9% in FamCov1 and 52.0% in FamCov2). FamCov1 included greater percentages of participants aged 45–54 and 55–64 years, greater percentage with tertiary education, greater percentage with no income and with higher monthly household income of HK$30,000 or above, and greater percentage living in owned housing than FamCov2. The differences, though statistically significant, were of very small or small effect size. FamCov1 had a lower percentage (15.1% vs. 22.5%) of participants with very low socioeconomic status, but a higher percentage (34.0% vs. 29.1%) with medium socioeconomic status than FamCov2. The differences in SES were statistically significant, with small effect size.

**Table 1:** Sociodemographic characteristics of participants of FamCov1 (May 2020) and FamCov2 (March 2021) surveys.

	**FamCov1**		**FamCov2**		**Cohen's**	
**Variables**	**Unweighted** ***n* (%)**	**Weighted** ***n* (%)**	**Unweighted** ***n* (%)**	**Weighted** ***n* (%)**	**W** ** (weighted)**	**P** ** (weighted)**
Sex						
Male	2,138 (43.7)	2,302 (47.1)	3,001 (50.2)	2,866 (48.0)	0.01	0.35
Female	2,753 (56.3)	2,589 (52.9)	2,973 (49.8)	3,108 (52.0)		
Age						
18–24	219 (4.5)	372 (7.6)	488 (8.2)	529 (8.9)	0.16	<0.001
25–34	1,090 (22.3)	658 (13.5)	1,306 (21.9)	932 (15.6)		
35–44	1,359 (27.8)	877 (17.9)	1,418 (23.7)	1,028 (17.2)		
45–54	1,204 (24.6)	1,076 (22.0)	1,343 (22.5)	1,112 (18.6)		
55–64	809 (16.5)	1,119 (22.9)	1,067 (17.9)	1,202 (20.1)		
65 or above	210 (4.3)	788 (16.1)	352 (5.9)	1,170 (19.6)		
Education						
Secondary or below	659 (13.6)	3,021 (62.2)	1,098 (18.5)	3,847 (64.9)	0.03	0.004
Tertiary	4,199 (86.4)	1,837 (37.8)	4,830 (81.5)	2,083 (35.1)		
Household income						
(HK$ monthly)						
No income	303 (6.8)	451 (10.1)	243 (4.7)	412 (7.8)	0.13	<0.001
9,999 or below	123 (2.8)	246 (5.5)	176 (3.4)	331 (6.2)		
10,000–19,999	363 (8.2)	700 (15.7)	551 (10.6)	935 (17.6)		
20,000–29,999	507 (11.4)	734 (16.4)	679 (13.1)	970 (18.3)		
30,000–39,999	576 (12.9)	687 (15.4)	691 (13.3)	749 (14.1)		
40,000 or above	2,579 (57.9)	1,651 (36.9)	2,849 (54.9)	1,903 (35.9)		
Housing type						
Rented	1,603 (33.9)	1,732 (36.3)	2,205 (37.0)	2,385 (40.0)	0.04	<0.001
Owned	3,120 (63.8)	3,040 (63.7)	3,758 (63.0)	3,580 (60.0)		
Socioeconomic status						
Very low	134 (3.2)	627 (15.1)	322 (6.2)	1,188 (22.5)	0.17	<0.001
Low	656 (15.7)	1,416 (34.1)	1,022 (19.8)	1,768 (33.5)		
Medium	1,497 (35.8)	1,409 (34.0)	1,915 (37.0)	1,539 (29.1)		
High	1,891 (45.3)	689 (16.8)	1,912 (37.0)	786 (14.9)		

Missing data were excluded. US$1 = HK$7.8. Socioeconomic status was defined by a composite score of education, household monthly income per person, and housing, analyzed as very low (0), low (1), medium (2) and high (3). Weighting was applied based on the distribution of sex, age, and education in the 2020 Hong Kong population. Cohen's W for effect size (difference between 2 surveys): 0.10, small; 0.30, medium; 0.50, large.

### Change in prevalence of individual perceived benefits and harms from FamCov1 to FamCov2

Table 2 shows that all but one (17 out of 18) perceived benefits showed significant increase in prevalence from FamCov1 to FamCov2. The largest increases were increased rest time, increased personal time, improved personal hygiene, reduced cold and flu, and increased anti-epidemic knowledge, and increased family time and increased family hygiene. Out of 11 perceived harms, 6 showed significant decreases and 4 showed significant increases, all with small effect size, except for the largest increase in decreased family time with medium effect size.

**Table 2:** Sex- and age-adjusted weighted prevalence (95% confidence intervals) of perceived benefits and harms in FamCov1 (May 2020) and FamCov2 (March 2021) surveys.

**Variables**	**FamCov1**	**FamCov2**	**Prevalence ratio (PR)** ** (FamCov2/** **FamCov1)**	**Partial eta squared**	**P**
	**Prevalence** ** (95% CI)**	**Prevalence** ** (95% CI)**			
**Perceived personal benefits**
Physical
Improved overall health	8.4 (7.3, 9.4)	17.9 (16.9, 18.8)	2.1	0.019	<0.001
Improved personal hygiene	18.6 (17.1, 20.0)	56.9 (55.6, 58.1)	3.1	0.149	<0.001
Reduced cold and flu	10.5 (9.1, 11.9)	51.3 (50.2, 52.5)	4.8	0.184	<0.001
Increased anti-epidemic knowledge	19.8 (18.3, 21.3)	56.9 (55.6, 58.1)	2.9	0.139	<0.001
Psychological
Decreased negative emotions	1.9 (1.5, 2.4)	2.2 (1.8, 2.6)	1.2	0.000	0.46
Increased positive emotions	4.1 (3.3, 4.8)	6.3 (5.7, 6.9)	1.5	0.002	<0.001
Increased adversity coping capability	10.9 (9.7, 12.1)	20.7 (19.7, 21.7)	1.9	0.017	<0.001
Social
Increased work/study efficiency	4.7 (3.9, 5.4)	6.9 (6.2, 7.5)	1.5	0.002	<0.001
Increased rest time	0.4 (−0.7, 1.4)	25.6 (24.7, 26.5)	64.0	0.124	<0.001
Increased personal time	0.5 (−0.6, 1.6)	27.8 (26.8, 28.7)	55.6	0.135	<0.001
**Perceived family benefits**
Physical
Improved family health	10.1 (8.9, 11.3)	24.4 (23.3, 25.4)	2.4	0.034	<0.001
Improved family hygiene	16.8 (15.4, 18.2)	49.9 (48.7, 51.2)	3.0	0.118	<0.001
Psychological
Decreased family negative emotions	1.9 (1.4, 2.4)	3.3 (2.9, 3.8)	1.7	0.002	<0.001
Increased family positive emotions	5.4 (4.6, 6.2)	8.0 (7.3, 8.7)	1.5	0.003	<0.001
Increased family happiness	3.9 (3.2, 4.6)	5.6 (5.0, 6.2)	1.4	0.002	<0.001
Increased family harmony	7.0 (6.2, 7.9)	9.1 (8.3, 9.8)	1.3	0.001	<0.001
Increased family adversity coping capability	9.8 (8.6, 10.9)	19.7 (18.7, 20.7)	2.0	0.019	<0.001
Social
Increased family time	0.7 (−0.6, 1.9)	51.3 (50.3, 52.4)	73.3	0.304	<0.001
**Perceived personal harms**
Physical
Delays in doctor consultation	9.9 (8.9, 11.0)	14.0 (13.1, 15.0)	1.4	0.004	<0.001
Psychological					
Caused depression	14.0 (13.0, 15.1)	11.3 (10.4, 12.2)	0.8	0.002	<0.001
Caused anxiety	33.5 (32.0, 34.9)	24.1 (22.8, 25.3)	0.7	0.011	<0.001
Increased negative emotions	43.3 (41.7, 44.8)	39.7 (38.3, 41.0)	0.9	0.001	0.001
Social					
Decreased work/study efficiency	15.8 (14.6, 17.0)	19.2 (18.2, 20.2)	1.2	0.002	<0.001
**Perceived family harms**					
Psychological					
Increased family negative emotions	32.6 (31.2, 33.9)	18.6 (17.4, 19.8)	0.6	0.026	<0.001
Decreased family happiness	18.7 (17.5, 19.9)	15.4 (14.3, 16.4)	0.8	0.002	<0.001
Decreased family harmony	12.0 (11.0, 13.1)	12.3 (11.4, 13.2)	1.0	0.000	0.68
Social					
Increased family conflicts	13.1 (12.0, 14.3)	18.9 (17.9, 19.9)	1.4	0.006	<0.001
Decreased family income	38.6 (37.0, 40.1)	35.1 (33.8, 36.4)	0.9	0.001	<0.001
Decreased family time	0.3 (−0.7, 1.4)	24.3 (23.4, 25.2)	81.0	0.120	<0.001

Missing data were excluded. Weighting was applied based on the distribution of sex, age, and education in the 2020 Hong Kong population. A multivariate model of analysis of covariance with sex and age entered as covariates were used for significance testing. Partial eta squared for effect size (difference between 2 surveys): 0.01, small; 0.06, medium; 0.14, large.

### Socioeconomic disparities in the total number of perceived benefits and harms in FamCov1 and FamCov2

**Figure 2:**
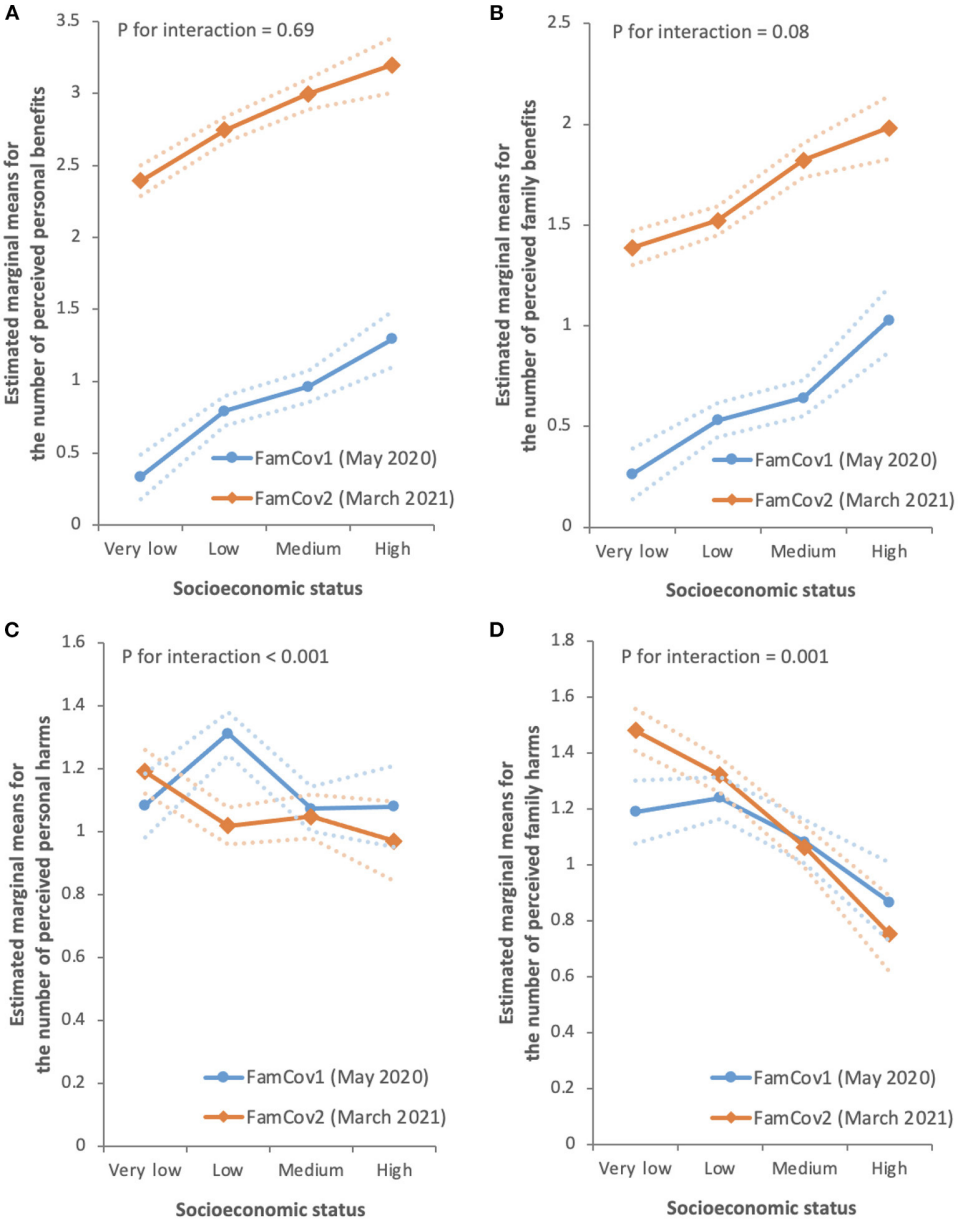
Sex- and age-adjusted estimated marginal means for the number of perceived personal benefits **(A)**, family benefits **(B)**, personal harms **(C)**, and family harms **(D)** by socioeconomic status (SES) for each survey. The error bands indicate the upper and lower bounds of the 95% confidence intervals. Socioeconomic status was defined by a composite score of education, household monthly income per person, and housing, analyzed as very low (0), low (1), medium (2) and high (3). Weighting was applied based on the distribution of sex, age, and education in the 2020 Hong Kong population.

Table 3 and Figure 2 show that the total number of perceived personal and family benefits significantly increased greatly (by almost 2-fold) from FamCov1 to FamCov2 (from 0.85 to 2.83 and 0.62 to 1.68, respectively; all *P* < 0.001), and increased with higher socioeconomic status (in both surveys all *P* for trend < 0.001). The magnitude (slope) of such socioeconomic disparities in FamCov1 and FamCov2 was similar (*P* for interactions = 0.69 and 0.08, respectively). For perceived personal harms, the total number significantly but slightly decreased (by 7%) from 1.14 in FamCov1 to 1.06 in FamCov2 (*P* = 0.01), with a significant linear trend in FamCov2 (*P* for trend = 0.005) but not in FamCov1 (*P* for trend = 0.25; *P* for interaction < 0.001). As for perceived family harms, in both surveys, the total numbers were similar and decreased with higher socioeconomic status (all *P* for trend < 0.001). Such socioeconomic disparities were greater in FamCov2, as shown by the greater decrease (from 1.48 to 0.75) in FamCov2 than that in FamCov1 (from 1.19 to 1.24 to 1.08 and 0.87; *P* for interaction = 0.001).

**Table 3:** Weighted sex- and age-adjusted estimated marginal means (95% confidence intervals) and the multivariate model of analysis of covariance for the total number of perceived personal benefits, family benefits, personal harms, and family harms in FamCov1 (May 2020) and FamCov2 (March 2021) surveys.

**Variables**	**Survey (Time)**	**Overall**	**Socioeconomic status (SES)**				**P for linear trend**	**P for interaction**
			**Very low**	**Low**	**Medium**	**High**		
Personal benefits	FamCov2 (March 2020)	2.83	2.39	2.75	3.00	3.20	<0.001	0.69
		(2.77, 2.90)	(2.29, 2.50)	(2.66, 2.83)	(2.89, 3.10)	(3.01, 3.39)		
	FamCov1 (May 2021)	0.85	0.34	0.79	0.96	1.29	<0.001	
		(0.77, 0.92)	(0.18, 0.49)	(0.69, 0.90)	(0.86, 1.07)	(1.10, 1.49)		
	Difference	1.99	2.05	1.95	2.03	1.9		
	(95% CI for difference)	(1.89, 2.08)	(1.87, 2.24)	(1.82, 2.09)	(1.88, 2.18)	(1.63, 2.18)		
	*P* for difference	<0.001	<0.001	<0.001	<0.001	<0.001		
Family benefits	FamCov2 (March 2020)	1.68	1.39	1.52	1.82	1.98	<0.001	0.08
		(1.63, 1.73)	(1.30, 1.47)	(1.45, 1.59)	(1.74, 1.91)	(1.83, 2.14)		
	FamCov1 (May 2021)	0.62	0.26	0.53	0.64	1.03	<0.001	
		(0.56, 0.67)	(0.14, 0.39)	(0.45, 0.62)	(0.55, 0.73)	(0.87, 1.19)		
	Difference	1.06	1.12	0.99	1.18	0.95		
	(95% CI for difference)	(0.98, 1.14)	(0.97, 1.27)	(0.88, 1.10)	(1.06, 1.30)	(0.73, 1.18)		
	P for difference	<0.001	<0.001	<0.001	<0.001	<0.001		
Personal harms	FamCov2 (March 2020)	1.06	1.19	1.02	1.05	0.97	0.005	<0.001
		(1.01, 1.10)	(1.12, 1.26)	(0.96, 1.08)	(0.98, 1.12)	(0.84, 1.10)		
	FamCov1 (May 2021)	1.14	1.08	1.31	1.07	1.08	0.249	
		(1.09, 1.19)	(0.98, 1.18)	(1.24, 1.38)	(1.00, 1.14)	(0.95, 1.21)		
	Difference	−0.08	0.11	−0.29	−0.02	−0.11		
	(95% CI for difference)	(−0.14, −0.02)	(−0.02, 0.23)	(−0.38, −0.21)	(−0.12, 0.07)	(−0.29, 0.07)		
	P for difference	0.01	0.09	<0.001	0.63	0.23		
Family harms	FamCov2 (March 2020)	1.16	1.48	1.32	1.06	0.75	<0.001	0.001
		(1.11, 1.20)	(1.41, 1.56)	(1.26, 1.38)	(0.99, 1.14)	(0.62, 0.89)		
	FamCov1 (May 2021)	1.10	1.19	1.24	1.08	0.87	<0.001	
		(1.04, 1.15)	(1.08, 1.30)	(1.16, 1.31)	(1.00, 1.16)	(0.73, 1.01)		
	Difference	0.06	0.29	0.08	−0.02	−0.12		
	(95% CI for difference)	(−0.01, 0.13)	(0.16, 0.43)	(−0.02,.0.18)	(−0.13, 0.09)	(−0.31, 0.08)		
	P for difference	0.10	<0.001	0.11	0.74	0.10		

Missing data were excluded. Sex and age were entered as covariates. Socioeconomic status was defined by a composite score of education, household monthly income per person, and housing, analyzed as very low (0), low (1), medium (2) and high (3). Weighting was applied based on the distribution of sex, age, and education in the 2020 Hong Kong population.

## Discussion

We have first reported that the prevalence of perceived benefits, both personal and family, of the COVID-19 outbreak substantially increased over a period of about 10 months (from May 2020 [FamCov1] to February and March 2021 [FamCov2]), the prevalence of perceived harms showed no substantial decrease, and socioeconomic disparities in both the total number of perceived personal and family benefits and the total number perceived personal and family harms. People of higher socioeconomic status reported more perceived benefits, whereas those of lower socioeconomic status reported more perceived harms. Despite the substantial increase in the total number of perceived personal and family benefits from FamCov1 to FamCov2, the socioeconomic disparities persisted over time.

Remarkably, the substantial increase in the total number of perceived personal and family benefits over time occurred without any notable improvements in the COVID-19 situation in Hong Kong. In fact, Figure 1 shows that the daily number of confirmed cases was greater before FamCov2 than before FamCov1. Thus, the large unexpected increase in the total number of perceived benefits was unlikely due to improving outbreak control and socioeconomic environment in Hong Kong.

It is plausible that people's perception of the COVID-19 pandemic and related problems had changed over time. Previous studies showed that a small, non-zero amount of past life adversity was associated with higher life satisfaction and appreciation of positive events in the present (39–41). We have reported earlier about fear, anxiety, and depression during early COVID-19 outbreaks in Hong Kong (7, 8, 36). Subsequent successful control of each outbreak through government policies and community collective efforts with low infection, severe COVID-19 disease and death rates might have led to increased confidence and self-efficacy in the population. Hong Kong had almost 100% voluntary masking, which became mandatory in July 2020 after 6 months from the beginning of Wave 1. Also, Hong Kong has no lockdown so far, which means people's freedom of movement and related livelihood are not severely limited, as they wear a mask in public places. Experience from coping and overcoming adversity might have helped people to better build resilience and appreciate some new or small pleasures in life amid the evolving pandemic. However, reports on these were lacking. One mechanism by which positive outcomes occur after negative experience could be cognitive reappraisal, a type of emotional regulation strategy where people perceived a stressful situation as not just a negative threat but rather a positive challenge (42). Cognitive reappraisal has been shown to be associated with reduced perceived stress and anxiety symptoms in isolated people amid COVID-19 in Hong Kong (31). Research to increase perceived benefits by designing and testing cognitive reappraisal interventions for improving mental health and well-being of the general population is warranted.

We found the greatest increase in perceived personal and family benefits in the physical and social aspects related to the pandemic. Particularly, under the physical category, improved personal hygiene, reduced cold and flu, increased anti-epidemic knowledge, and improved personal and family hygiene all showed the largest increases. For the social category, increased rest time, increased personal and family time also showed large increases. Most other perceived benefits under the psychological category, such as those concerning emotions and adversity coping capability all showed only modest increases. These suggest that physical and social benefits were not proportionately accompanied by emotional benefits in most people, as the pandemic is so strongly linked to negative feelings or affect. Special actions such as counseling and psychosocial support are needed to tackle emotional problems in those who are most vulnerable. On the other hand, the prevalence of increased personal and family adversity coping capability after Waves 1 and 2 (in FamCov1) which doubled after Waves 3 and 4 (in FamCov2) could be mainly due to the greater increases in perceived physical and social benefits, as adversity coping capability indicates self-efficacy rather than emotion.

In contrast to the many great increases in perceived benefits in FamCov2 from small prevalence in FamCov1, we found that prevalence of perceived harms in FamCov1 were much higher, and perceived harms of COVID-19 outbreak changed in mixed directions and with no substantial decreases. This pattern suggests that increase in perceived benefits do not necessarily reduce perceived harms. This is expected as the pandemic was evolving, control measures were still stringent, and the threat of COVID-19 infection, isolation and quarantine, and severe illness, and the severe restrictions of cross boarder travels, were still on-going. Wave 4 was just brought under control during FamCov2 and most other countries continued to have serious outbreaks. Whether perceived harms (which remained stable) and perceived benefits (which had increased) would change further, with the much more serious Wave 5 outbreak started in Hong Kong in February–March 2022 and appeared under control in April–May 2022, should be further studied.

For individual items, the two specific items which showed the greatest change were the harms of decreased family time (from 0.3 to 24.3%) and the benefits of increased family time (0.7–51.3%). Such apparently conflicting results of increased harms and benefits in the same surveys could be due to opposite consequence from the same social distancing measures on people of different socioeconomic status. Those with more socioeconomic resources or adversity coping capability were more likely to benefit from work-from-home and school closure and enjoy more quality family time than those who were under-privileged. Further research on such disparities is warranted.

Of interest, our results showed that both the perceived harms items on depression and anxiety decreased significantly. This corroborates the results of a previous longitudinal observation cohort study in England that the levels of depression and anxiety declined over time during the COVID-19 pandemic (30), as well as that of a study using repeated cross-sectional surveys in Hong Kong which reported declines in both probable depression and anxiety from February to August 2020 (9). The above results collectively suggest that some people had adapted to the adverse situation amid the COVID-19 pandemic over time.

A particularly interesting finding of our study was that the socioeconomic disparities in the perceived personal and family benefits of COVID-19 outbreak persisted despite a substantial increase in total numbers of perceived benefits over time. It has been reported previously that the COVID-19 pandemic affected the worldwide population with significant socioeconomic disparities (12–16). Socioeconomic differences have been observed in various domains, such as income, happiness, behavior, and health (32–34, 43, 44). It has been hypothesized that two comparative processes determine subjective well-being as it relates to socioeconomic status: (1) comparison to one's recent past experience, and (2) social comparison between individuals (32, 33). According to this framework, the initial waves of COVID-19 undermined one's health and well-being more strongly because the comparison was made with an earlier normal period where the larger environment was relatively safe and secure. The discrepancy between the current experience (the initial waves of outbreak) and recent past experience (a relatively low infection risk environment) created an unexpected shock. However, as the pandemic continued, the past experience (the initial waves of outbreak) against which the later experience (subsequent waves of outbreak) was compared with would make the latter seem less surprising or shocking. Thus, people would further adapt to the “new normal” situation and regain a sense of health and well-being amid the pandemic especially when a more serious outbreak had been controlled.

While we found an overall improvement in perceived benefits with time, their socioeconomic inequalities persisted. The latest available Gini coefficient of Hong Kong based on original household income was 0.539 in 2016, increased from 0.518 in 1996 and 0.533 in 2006. The coefficients were amongst the highest in the world. The increased socioeconomic disparities in Hong Kong as a whole suggests that any benefits and adaptation that had occurred were primarily due to the repeated experiences with COVID-19 outbreaks, but not so much from a substantial improvement in the disparities in individuals' socioeconomic situations. Further, the present data indicated that the linear trends of the socioeconomic gradients for perceived harms were more pronounced in FamCov2 than in FamCov1. Thus, in terms of perceived harms, socioeconomic disparities had modestly increased over time in the present study. Our results suggest that socioeconomic disparities in the harms of COVID-19 might continue to expand as the pandemic continued and forewarn that Hong Kong's Gini coefficient would get worse. Interventions to reduce disparities related to COVID-19 and beyond are urgently needed and future changes should be monitored and studied.

Two methodological issues should be addressed. First, the list of items in the two surveys were similar but not identical. New and modified items were used in FamCov2 in response to the evolving pandemic, and corresponding public health measures. However, comparisons were only made for common items across both surveys. Another methodological issue concerns with the validity of the tools used to measure perceived benefits and harms. We have previously reported that people who selected a greater total number of perceived benefits reported higher levels of happiness and decreased drinking during the COVID-19 pandemic, whereas those who selected a greater total number of perceived harms reported lower happiness and increased drinking (37). In previous (21) and present results, we also found that socioeconomic status was positively related to perceived benefits but negatively related to perceived harms. Thus, there is some evidence of convergent validity with our measures of perceived benefits and harms. However, further work is needed to provided other forms of validity, including those that discriminate between personal and family benefits and harms.

The present study had some other limitations. First, perceived benefits and harms were self-reported and might be subject to errors. However, objective measures of harms and benefits are impracticable amid the pandemic. Second, to minimize the length of the questionnaire, we did not ask about the intensity of the benefits and harms and the details of each benefit/harm. Third, as the present data were collected from two cross-sectional surveys separated by an interval of 10 months, and the participants were different, whether the temporal changes and socioeconomic disparities could apply to the same individuals over time is uncertain. Prospective follow-up studies on the same cohort would be needed to measure changes in individuals and the predictive factors. Fourth, although the differences in sociodemographic characteristics of the participants in the two surveys were statistically significant because of the large sample size, the effect size of the differences were quite small. Because in both surveys, we aimed to collect data from as many respondents as possible within a short period so that the results would not be affected by sudden increase of COVID-19 cases during the data collection period, the response rates were low, and the generalizability of our results might have been limited.

In conclusion, we have first reported substantial increases in perceived personal and family benefits over harms of the COVID-19 outbreak in the adult population over 10 months amid the COVID-19 pandemic, and despite such large increases in perceived personal and family benefits, their socioeconomic disparities remained unchanged. Further, although the perceived harms of the COVID-19 outbreak remained quite stable overall, their socioeconomic disparities modestly increased. Urgent interventions are needed to address the unequal impacts of COVID-19 on individuals and families of different socioeconomic status and to reduce socioeconomic disparities. We recommend that more COVID-19 relief efforts should be directed to people of lower socioeconomic status, and that public health education as well as mental health support should be provided at the community level. Various harms and benefits and their socioeconomic disparities in the general population amid the COVID-19 pandemic need to be monitored regularly and addressed urgently.

## Data availability statement

The dataset presented in this article is not readily available because the sharing of data to third parties was not mentioned in the subjects' consent. Requests to access the dataset can be directed to the corresponding author/s.

## Ethics statement

The studies involving human participants were reviewed and approved by Ethics approval was obtained from the Institutional Review Board of the University of Hong Kong/Hospital Authority Hong Kong West Cluster (IRB reference no.: UW20-238). The patients/participants provided their written informed consent to participate in this study.

## Author contributions

Conceptualization and project administration: HL, TL, SH, and MW. Methodology: TL, AL, SH, and MW. Validation, formal analysis, and data curation: HL and WG. Investigation and writing—original draft preparation: HL and TL. Resources, supervision, and funding acquisition: TL, SH, and MW. Writing—review and editing: HL, TL, WG, SS, AL, SH, and MW. Visualization: HL, TL, and SH. All authors contributed to the article and approved the submitted version.

## Funding

This research was funded by the Hong Kong Jockey Club Charities Trust.

## Conflict of interest

The authors declare that the research was conducted in the absence of any commercial or financial relationships that could be construed as a potential conflict of interest.

## Publisher's note

All claims expressed in this article are solely those of the authors and do not necessarily represent those of their affiliated organizations, or those of the publisher, the editors and the reviewers. Any product that may be evaluated in this article, or claim that may be made by its manufacturer, is not guaranteed or endorsed by the publisher.
